# Combining Shapley value and statistics to the analysis of gene expression data in children exposed to air pollution

**DOI:** 10.1186/1471-2105-9-361

**Published:** 2008-09-02

**Authors:** Stefano Moretti, Danitsja van Leeuwen, Hans Gmuender, Stefano Bonassi, Joost van Delft, Jos Kleinjans, Fioravante Patrone, Domenico Franco Merlo

**Affiliations:** 1 Epidemiology and Biostatistics, National Cancer Research Institute, Genova, Italy; 2 Department of Health Risk and Toxicology, Maastricht University, Maastricht, the Netherlands; 3 Genedata AG, Basel, Switzerland; 4 Unit of Molecular Epidemiology, National Cancer Research Institute, Genova, Italy; 5 DIPTEM, University of Genova, Genova, Italy

## Abstract

**Background:**

In gene expression analysis, statistical tests for differential gene expression provide lists of candidate genes having, individually, a sufficiently low *p*-value. However, the interpretation of each single *p*-value within complex systems involving several interacting genes is problematic. In parallel, in the last sixty years, *game theory *has been applied to political and social problems to assess the power of interacting agents in forcing a decision and, more recently, to represent the relevance of genes in response to certain conditions.

**Results:**

In this paper we introduce a Bootstrap procedure to test the null hypothesis that each gene has the same relevance between two conditions, where the relevance is represented by the Shapley value of a particular coalitional game defined on a microarray data-set. This method, which is called *Comparative Analysis of Shapley value *(shortly, CASh), is applied to data concerning the gene expression in children differentially exposed to air pollution. The results provided by CASh are compared with the results from a parametric statistical test for testing differential gene expression. Both lists of genes provided by CASh and t-test are informative enough to discriminate exposed subjects on the basis of their gene expression profiles. While many genes are selected in common by CASh and the parametric test, it turns out that the biological interpretation of the differences between these two selections is more interesting, suggesting a different interpretation of the main biological pathways in gene expression regulation for exposed individuals. A simulation study suggests that CASh offers more power than t-test for the detection of differential gene expression variability.

**Conclusion:**

CASh is successfully applied to gene expression analysis of a data-set where the joint expression behavior of genes may be critical to characterize the expression response to air pollution. We demonstrate a synergistic effect between coalitional games and statistics that resulted in a selection of genes with a potential impact in the regulation of complex pathways.

## Background

Microarray technology allows for the simultaneous detection of expression levels of thousands of genes. By means of gene expression microarrays it is possible to generate a matrix of expression data, where the rows index the genes and the columns the study samples. Numbers in the matrix represent gene expression values in the study samples. Many statistical methods have been proposed for the selection of candidate genes that potentially play an important role in the mechanisms governing the biological system [1-3].

Unfortunately, the main difficulty in choosing which statistical approach to use is that most methods are not directly related with a sound biological interpretation. For example, statistical testing [1,4,5] for gene selection aims at finding genes which are 'strongly' differentially expressed between two conditions, where for *condition *we mean whatever state of the biological samples that is conjectured to affect gene expression (e.g. the exposure to environmental or therapeutic agents, disease state, etc.). Following this approach, genes are usually ranked according to their *p*-values, being genes with the smallest *p*-values the most differentially expressed. Since no biological meaning is necessarily associated to the notion of *p*-value, the interpretation of single *p*-values within complex biological systems where several genes are known to interact is problematic. For instance, a crucial issue to address is whether a subset of genes identified as being individually differentially expressed in the study samples is more or less efficient in characterizing samples than a subset of genes which show different levels of interaction between the two conditions.

A method for gene expression analysis based on game theory was proposed in [6] and is further explored in this paper. The main advantage of the game theory approach is the possibility to compute a numerical index, i.e. a *relevance index*, which represents the relevance of each gene under a certain condition taking into account the expression behaviors of the other genes under the same condition. An additional feature of the game theory approach developed in [6] is that it is provided a novel property driven characterization of the Shapley value in order to contextualize and justify the use of the Shapley value as relevance index for genes. Five simple properties with a biological interpretation are introduced in [6] and it is proved that they characterize the Shapley value. One simple property is that a relevance index should attribute null relevance to genes that are never up- or down- regulated under a certain condition. This idea is captured by the Null Gene (NG) property. In addition, if one is interested to bring smaller gene pathways into prominence, then another reasonable property is that if two disjoint sets of genes are up- or down-regulated in a same rate of samples, then genes in the smaller set should receive a higher relevance index than genes in the bigger one (Partnership Monotonicity (PM)). The Partnership Rationality (PR) property and the Partnership Feasibility (PF) property determine, respectively, a lower and an upper bound of the power of certain pathways of genes in determining the onset of the tumor. Lastly, it is used a special version of additivity, namely the Equal Splitting (ES) property, which has the natural interpretation of giving the same reliability to different microarray experiments. It is proved in [6] that the Shapley value is the unique relevance index which satisfies the properties PR, PF, PM, ES and NG on the class of microarray games. We refer to [6] for a more detailed description and discussion of such properties.

According to the game theory approach, the frequency of *associations *(see *Methods*) of all of the subsets of genes with a condition of interest is described by means of a *microarray game*. The definition of relevance index for genes is provided in terms of the *Shapley value *[7,8], which is the unique relevance index for microarray games satisfying the set of properties introduced in [6]. The higher the number attributed by the Shapley value to a certain gene in a given microarray game, the higher the relevance of that gene for the mechanisms governing the genomic effects of the condition under study.

Since gene expression is a stochastic, or 'noisy', process [9,10] and a microarray game is defined on a gene expression data-set, a microarray game itself follows a stochastic law, significantly affecting the stability of a relevance index. This fact must be considered in comparing the relevance index of genes under different conditions, e.g. different environmental exposures.

The purpose of this work is to introduce a new method to analyze gene expression data, which combines the game theory notion of relevance index [6] with the notion of statistical significance. A Bootstrap based algorithm applied to the sample statistics of the Shapley value is introduced in this paper and is used to perform a *Comparative Analysis of Shapley value *(shortly, CASh). CASh is used to select those genes whose relevance index results stable against noise in gene expression, meaning that the index has the tendency to be weakly affected when a few observations are removed. The basic idea of Bootstrap [11-13] is to use re-sampling techniques to collect information about the shape, center, and spread of the sampling distribution of the statistic of interest. This idea is particularly valuable when it is not possible to assume a given model describing the distributions in the population and to calculate the parameters of the corresponding sampling distribution.

To illustrate the framework's utility of the method, we applied CASh to gene expression data published in [14]. In [14] genome-wide oligonucleotide microarray analysis was applied in peripheral blood cells of children from Teplice (TP) area (n = 23), and compared with children from the rural control area of Prachatice (PR) (n = 24) in the Czech Republic. The TP area is a mining district characterized by high levels of airborne pollutants including carcinogens [14]. The results provided by CASh in this application are compared with the results provided by a parametric statistical analysis for the selection of differentially expressed genes between the two areas.

Other approaches using Game Theory for gene expression analysis have been proposed in literature. An approach explored in [15] is based on the framework of minimum cost spanning trees (MCST), that is used to represent the interactions between all possible pairs of genes and is extended to implement the notion of association for coalitions of genes. Basically, this approach is rooted on two main steps: first, a method based on the MCST problem is introduced to represent the interactions between the involved genes; second, the MCST representation of a gene expression dataset is used to analyze a related game theoretical problem for the determination of the relevance of genes. Another application introduced in [16] is related to the problems of making good prediction of sample conditions. Classification games are defined and used to analyze the power of groups of genes to classify samples into the right study conditions. Classification games turn out to be closely related to microarray games and, on some numerical examples, the Shapley value is studied as a method for selection of genes with high performance in sample classification. Recently, in [17], a set of genes selected according to the Shapley value is studied in connection with the pathogenesis of neuroblastic tumors.

Another approach to computational biology using game theory is the *multi-perturbation Shapley value analysis *(MSA) [18], that is a method for causal function localization which addresses the challenge of defining and calculating the contributions of network elements from a data set of multiple lesions or other type of perturbations and their corresponding performance scores. In this framework, a set of multiple lesion experiments is represented as a coalitional game. Specifically, MSA defines the set of contributions to be the Shapley value, which stands for the unique fair division of the game's worth (the network's performance score when all elements are intact) among the different players (the network elements). The contribution of an element to a function measures its importance, that is, the part it causally plays in the successful performance of that function. MSA has recently been used in analysis of data from genetic experiments in a work by [19]. The aim of the work by [19] was to identify the importance in terms of causal responsibility of some genes in performing a certain function in yeast cells. In their approach, [19] evaluate the value of each coalition as a measure of the biological system's performance for a certain function (*e*.*g*. the ability of the system to survive the UV irradiation).

## Results

### Model application

**Figure 1:**
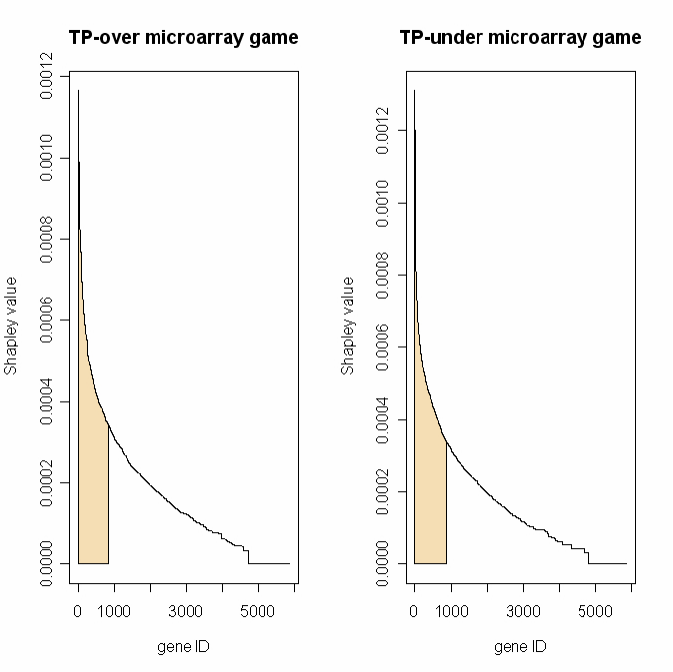
**Shapley value plot**. (left side) Shapley value plot in game ${\bar{v}}^{TP+}$ (also called TP-over microarray game). (right side) Shapley value plot in game ${\bar{v}}^{TP-}$ (also called TP-under microarray game). Shapley values are shown in decreasing order. Genes with Shapley value greater than the mean plus the standard deviation are shown in the colored intervals (precisely, 838 genes in game ${\bar{v}}^{TP+}$ and 889 genes in game ${\bar{v}}^{TP-}$).

We applied CASh to the analysis of gene expression data published in [14] of 23 children from the polluted area of Teplice (TP) and 24 children from the rural control area of Prachatice (PR), in the Czech Republic. We addressed the problem of quantifying the relevance of genes in the TP area using the information provided by the microarray game defined when up-regulated genes are considered (${\bar{v}}^{TP+}$) and the microarray game defined when down-regulated genes are considered (${\bar{v}}^{TP-}$). The relevance of genes was computed as the Shapley values of games ${\bar{v}}^{TP+}$ and game ${\bar{v}}^{TP-}$ (see *Methods *for more computational details about CASh). The plot of the Shapley value distributions of genes in games ${\bar{v}}^{TP+}$ and ${\bar{v}}^{TP-}$ is shown in Figure 1 (for the formal definition of the notations used in this section see *Methods*).

Algorithm 1 was applied to compare the Shapley value *ϕ* of games ${\bar{v}}^{TP+}$ and ${\bar{v}}^{TP-}$ with the Shapley value computed on the microarray game defined when up-regulated genes in PR area are considered (${\bar{v}}^{PR+}$) and the Shapley value computed on the microarray game defined when down-regulated genes in PR area are considered (${\bar{v}}^{PR-}$), respectively. More precisely, Algorithm 1 was applied twice: first, to test the null hypothesis |*ϕ*_*i*_(${\bar{v}}^{TP+}$) - *ϕ*_*i*_(${\bar{v}}^{PR+}$)| = 0 against the alternative hypothesis |*ϕ*_*i*_(${\bar{v}}^{TP+}$) - *ϕ*_*i*_(${\bar{v}}^{PR+}$)|≠ 0; second, to test the null hypothesis |*ϕ*_*i*_(${\bar{v}}^{TP-}$) - *ϕ*_*i*_(${\bar{v}}^{PR-}$)| = 0 against the alternative hypothesis |*ϕ*_*i*_(${\bar{v}}^{TP-}$) - *ϕ*(${\bar{v}}^{PR-}$)| ≠ 0.

### Selection of significantly modified gene expressions in exposed versus non-exposed children

Genes were selected according to the double criterium of small *p*-value from CASh and large Shapley value in microarray games defined on TP data. More precisely, genes with an un-adjusted *p*-value, provided by Algorithm 1, lower than a predefined cut-off and Shapley value greater than the mean plus the standard deviation in ${\bar{v}}^{TP+}$ (838 genes; see Figure 1, left side) or ${\bar{v}}^{TP-}$ (889 genes; see Figure 1, right side), were selected (Table 1). The Shapley value in games defined on TP data represents a measure of the relevance of genes for the mechanisms governing the genomic effects of pollution in TP, whereas the Shapley value of microarray games defined on PR data is taken as a reference value. The latter is used in CASh to remove the effects of stochastic noise from the Shapley value of games defined on TP data. In *Methods: *Data processing for CASh, the procedure adopted to define all microarray games on the basis of the reference gene expression levels observed in PR is described.

**Table 1:** Analysis of real data with CASh and t-test.

	**CASh**			**t-test**			**CASh, t-test**
*p*-value	Genes	${\bar{v}}^{TP+}$	${\bar{v}}^{TP-}$	Genes	TP up	TP down	Intersection
			
< 0.0001	20	16	4	16	15	1	7
< 0.001	33	27	6	62	48	14	17
< 0.01	159	107	52	265	169	96	92
< 0.05	434	245	189	762	408	354	272

Overview of the number of genes with *p*-values lower than predefined cutoffs (first column). Columns 'Genes' show the total number of genes selected by CASh and t-test, respectively; column '${\bar{v}}^{TP+}$' shows the number of genes with Shapley value significantly different in microarray game ${\bar{v}}^{TP+}$ as compared to ${\bar{v}}^{PR+}$; column '${\bar{v}}^{TP-}$' shows the number of genes with Shapley value significantly different in microarray game ${\bar{v}}^{TP-}$ as compared to ${\bar{v}}^{PR-}$; column 'TP up' shows the number of genes up-regulated in children from TP as compared to children from PR; column 'TP down' shows the number of genes up-regulated in children from TP as compared to children from PR; column 'Intersection' shows the number of genes selected by both t-test and CASh at different levels of *p*-value.

**Figure 2:**
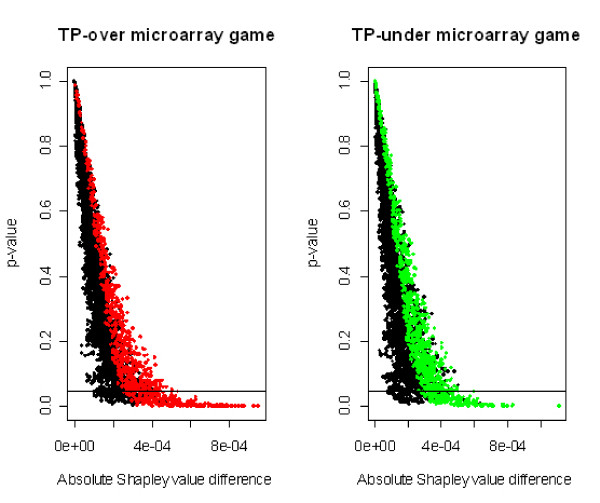
***p*-value vs. absolute Shapley difference**. Scatterplot of the *p*-values provided by the CASh method versus the absolute difference of Shapley values *abs*(*ϕ*(${\bar{v}}^{TP+}$) - *ϕ*(${\bar{v}}^{PR+}$)) (left side) and *abs*(*ϕ*(${\bar{v}}^{TP-}$) - *ϕ*(${\bar{v}}^{PR-}$)) (right side). Red points represent the 838 genes selected in game ${\bar{v}}^{TP+}$; green points represent the 889 genes selected in game ${\bar{v}}^{TP-}$.

Note that for each predefined value of *p *in Table 1, the number of genes selected in the microarray game ${\bar{v}}^{TP+}$ is larger than that selected in the microarray game ${\bar{v}}^{TP-}$. Figure 2 shows the scatterplots of the *p*-values versus the absolute differences in Shapley values. These plots indicates that the 838 genes selected in ${\bar{v}}^{TP+}$ and the 889 genes selected in ${\bar{v}}^{TP-}$ are closed to the respective Pareto optimal frontiers.

The numbers of genes selected by CASh and t-test for different levels of *p *are presented in Table 1, together with the number of genes which are selected by both methods. Table 2 shows the expression differences between individuals of the two regions of the 28 genes selected by CASh or t-test at *p *< 0.0001. Seven genes are selected by both methods.

**Table 2:** Description of genes selected by t-test or CASh (*n *= 28).

**ProbeID**	**Genbank Accession**	**Gene Symbol**	**GeneName**	**Effect**	**Selected by**
A_23_P154849	NM_138983	OLIG1	oligodendrocyte transcription factor 1	0,48	CASh
A_23_P35534	NM_020999	NEUROG3	neurogenin 3	0,28	CASh, t-test
A_23_P29248	NM_003312	TST	thiosulfate sulfurtransferase (rhodanese)	0,27	CASh, t-test
A_23_P412409	NM_015172	BAT2D1	BAT2 domain containing 1	-0,25	CASh, t-test
A_23_P382775	NM_014417	BBC3	BCL2 binding component 3	0,25	t-test
A_23_P122445	NM_005319	HIST1H1C	histone 1, H1c	0,25	CASh
A_23_P219060	NM_022107	GPSM3	G-protein signalling modulator 3 (AGS3-like, C. elegans)	0,24	t-test
A_23_P23194	NM_032409	PINK1	PTEN induced putative kinase 1	0,24	t-test
A_23_P69652	NM_080819	GPR78	G protein-coupled receptor 78	0,23	t-test
A_23_P154766	NM_080611	DUSP15	dual specificity phosphatase 15	0,22	CASh, t-test
A_23_P8293	AK093571	SCML4	sex comb on midleg-like 4 (Drosophila)	-0,22	CASh
A_23_P132285	NM_001013440	MPST	mercaptopyruvate sulfurtransferase	0,21	t-test
A_23_P38876	NM_005357	LIPE	lipase, hormone-sensitive	0,20	CASh
A_23_P357374	NM_178449	TIP39	tuberoinfundibular 39 residue protein precursor	0,20	CASh
A_23_P22487	NM_013271	PCSK1N	proprotein convertase subtilisin/kexin type 1 inhibitor	0,19	CASh, t-test
A_23_P143385	NM_004118	FKHL18	forkhead-like 18 (Drosophila)	0,19	t-test
A_23_P42991	*unknown*	*unknown*	*unknown*	0,18	CASh, t-test
A_23_P401524	NM_005205	COX6A2	cytochrome c oxidase subunit VIa polypeptide 2	0,17	CASh
A_23_P54330	NM_014691	AQR	aquarius homolog (mouse)	-0,17	t-test
A_23_P60520	U43747	FXN	frataxin	0,16	CASh, t-test
A_23_P57089	NM_020182	TMEPAI	transmembrane, prostate androgen induced RNA	0,16	CASh
A_23_P109837	NM_014850	SRGAP3	SLIT-ROBO Rho GTPase activating protein 3	0,16	t-test
A_23_P8571	NM_080744	SRCRB4D	scavenger receptor cysteine rich domain containing, group B (4 domains)	0,16	CASh
A_23_P109643	NM_172027	ABTB1	ankyrin repeat and BTB (POZ) domain containing 1	0,15	CASh
A_23_P57941	NM_005777	RBM6	RNA binding motif protein 6	-0,15	CASh
A_23_P130926	NM_017914	C19orf24	chromosome 19 open reading frame 24	0,13	CASh
A_23_P106641	NM_014329	RCD-8	autoantigen	-0,10	CASh
A_23_P127475	NM_005125	CCS	copper chaperone for superoxide dismutase	0,08	CASh

Expression changes of the 28 genes that were selected by t-test or CASh at *p*-value< 0.0001. 'Effect' column shows the difference between the average expression ratio in TP minus the average expression ratio in PR. In the last column, the method used for each gene selection is shown: t-test or CASh or both.

**Figure 3:**
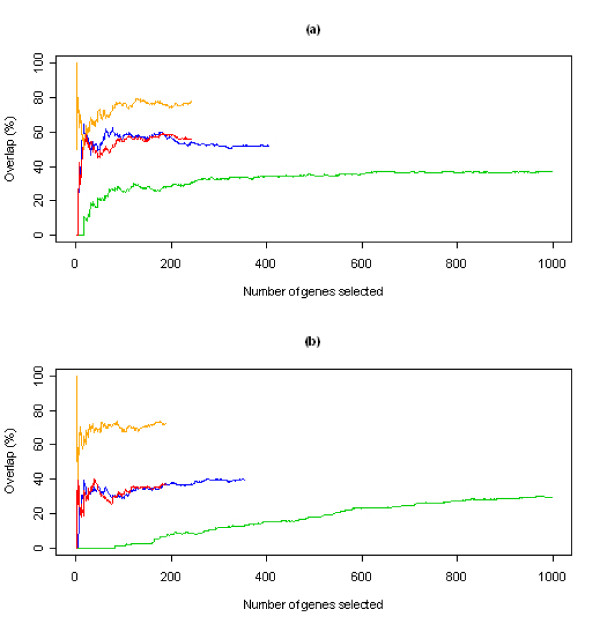
**Overlap curves**. Overlap of relevant gene lists generated using different selection criteria. The *x*-axis represents the number of genes selected as relevant genes, and the *y*-axis represents the overlap (%) of two gene lists for a given number of relevant genes. Figure (a) shows the overlap curves between different selection methods when the microarray game ${\bar{v}}^{TP+}$ is considered; Figure (b) shows the overlap between different selection methods when the microarray game ${\bar{v}}^{TP-}$ is considered. Green line: fold-change rank ordering versus absolute Shapley value difference rank ordering (for first 1000 genes ranked); blue line: *p*-value rank ordering (*p *< 0.05) on t-test versus Shapley value difference rank ordering; red line: *p*-value rank ordering (*p *< 0.05) on t-test versus *p*-value rank ordering (*p *< 0.05) on CASh; orange line: *p*-value rank ordering (*p *< 0.05) on CASh versus absolute Shapley value difference rank ordering.

The overlap between CASh and t-test in terms of the number of selected genes when ranked according to their *p*-values (*p *<0.05) in both CASh and t-test is represented in Figure 3 (red lines). Figure 3 also shows other three curves: the green line represents the overlap between a list of genes generated with the criterium of highest fold-change of average expression between TP and PR and a list of genes generated with the criterium of highest differential Shapley value between games on TP and PR; the blue line represents the overlap between a list of genes ranked according to their p-value (*p *< 0.05) in the t-test and a list of genes generated with the criterium of highest differential Shapley value between games on TP and PR; the orange line represents the overlap between a list of genes ranked according to their *p*-value (*p *< 0.05) in CASh and a list of genes generated with the criterium of highest differential Shapley value between games on TP and PR. The overlap between CASh *p*-values and t-test (red line) or between differential Shapley values and t-test (blue lines) is remarkable higher than the overlap between fold-changes and differential Shapley values (green lines). Using CASh instead of differential Shapley value does not determine any significant differences in terms of overlap with the t-test list.

**Figure 4:**
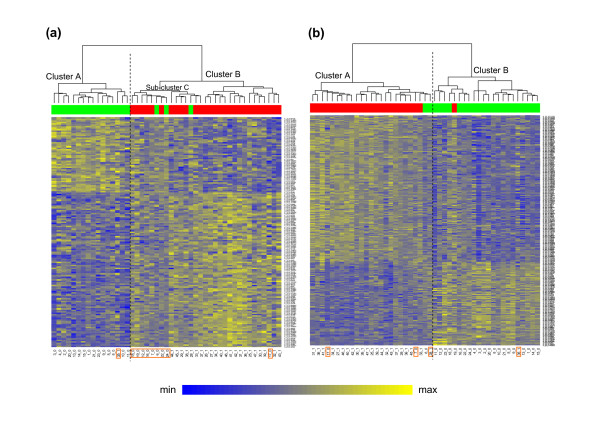
**Clustering on genes selected by CASh and t-test at *p *< 0.01**. (a) Heat-map of the log-expression values together with hierarchical clustering (Ward method, Euclidean distance) and K-means (a priori specified number of clusters *K *= 2) of 47 subjects (columns) and 159 genes (rows) with highest Shapley values and with un-adjusted *p*-values smaller than 0.01 (from CASh, Algorithm 1). (b) Heat-map of the log-expression values together with the hierarchical clustering (Ward method, Euclidean distance) and K-means (a priori specified number of clusters *K *= 2) of 47 subjects (columns) and 265 genes (rows) with *p*-values smaller than 0.01 (from t-test). Yellow: up-regulation and blue: down-regulation. In subject labels, 1 means exposed subject, whereas 0 means non-exposed subject. The red and green labels on the top of the heat-map represent the two clusters of subjects provided by *K*-means. Orange rectangles highlight misclassified subjects. The vertical dashed line shows the separation between the two main clusters.

The hierarchical clustering (Figure 4(a)) based on the set of 159 genes with highest Shapley value and unadjusted *p*-value < 0.01 returned a distinct separation of 22 (cluster B) exposed subjects and 15 (cluster A) non-exposed subjects (accuracy 78.7%). The hierarchical clustering (Figure 4(b)) based on the set of all of the 265 genes differentially expressed at *p *< 0.01 in the t-test returned a distinct separation of 22 (cluster A) exposed and 21 (cluster B) non-exposed subjects (accuracy 97.7%). The red/green bar on the top of the heat-maps is used to label the subjects of the two clusters provided by *K*-means clustering with *K *= 2. *K*-means clustering shows an accuracy of 88.4% for the list of genes selected by CASh and 97.7% for the list of genes selected by t-test.

**Figure 5:**
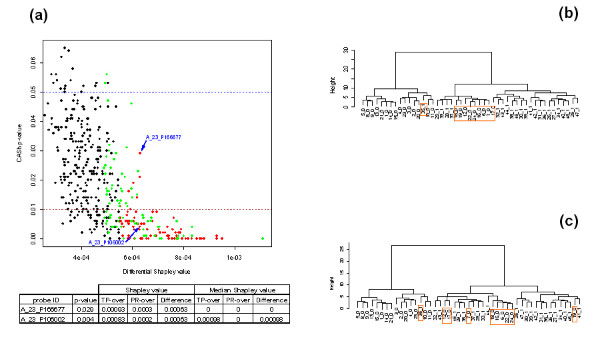
**CASh *p*-values versus differential Shapley value**. (a) Scatterplot of the *p*-values provided by the CASh method versus the differential Shapley values of the first 450 genes with the smallest *p*-value from CASh applied to ${\bar{v}}^{TP+}$*vs*. ${\bar{v}}^{PR+}$ and to ${\bar{v}}^{TP-}$*vs*. ${\bar{v}}^{PR-}$. Red points correspond to 80 genes selected with respect to the differential Shapley value *ϕ*(${\bar{v}}^{TP+}$) - *ϕ*(${\bar{v}}^{PR+}$). Green points correspond to 80 genes selected with respect to the differential Shapley value *ϕ*(${\bar{v}}^{TP-}$) - *ϕ*(${\bar{v}}^{PR-}$). The blue dashed line intercepts the *y*-axis in *p *= 0.05; The brown dashed line intercepts the *y*-axis in *p *= 0.01. Blue arrows indicate the two genes which are shown in the table (column 'Probe ID'), that are characterized by different *p*-values provided by CASh (column '*p*-value') but with the same Shapley value difference *ϕ*(${\bar{v}}^{TP+}$) - *ϕ*(${\bar{v}}^{PR+}$) = 0.00063 (see column 'Shapley value'). The medians of the statistics of the Shapley value in ${\bar{v}}^{TP+}$ and ${\bar{v}}^{PR+}$ together with the difference of the medians are shown in column 'Median Shapley value'. (b) Hierarchical clustering (Ward method, Euclidean distance) of 47 subjects (columns) on 160 genes selected according to the differential Shapley value (green and red points in (a)). (c) Hierarchical clustering (Ward method, Euclidean distance) of 47 subjects (columns) on 113 genes selected from the list of 160 genes with the highest differential Shapley value having a *p*-value from CASh lower than 0.01 (green and red points in (a) below the brown dashed line). In subject labels, 1 means exposed subject, whereas 0 means non-exposed subject. Orange rectangles highlight misclassified subjects.

Figure 5(a) shows that most of the 160 genes with highest Shapley value difference have also a small *p*-value: 37 genes out of these 160 have a *p*-value from CASh bigger than 0.01, and only 2 have a *p*-value bigger then 0.05. Blue arrows show two genes, precisely *A*_23_*P*166677 (MFSD1, major facilitator superfamily domain containing 1) and *A*_23_*P*106002 (NFKBIA, nuclear factor of kappa light polypeptide gene enhancer in B-cells inhibitor, alpha), with the same Shapley value difference between ${\bar{v}}^{TP+}$ and ${\bar{v}}^{PR+}$ of 0.0063, and very different *p*-values of 0.029 and 0.004, respectively. As it is shown in the table of Figure 5(a), for gene *A*_23_*P*166677 the difference between the medians of the sample statistics of the Shapley value in ${\bar{v}}^{TP+}$ and ${\bar{v}}^{PR+}$ is null, whereas the analogue difference of medians for gene *A*_23_*P*106002 is 0.00098. The mean of the sample statistic of the Shapley value in a microarray game equals the Shapley value of the game [see Additional file 1]. Differently, the median of the sample statistic of the Shapley value of a microarray game has not an immediate game theoretical interpretation but it is more stable than the mean with respect to exceptional values.

Figure 5(b) shows that the list of 160 genes with the highest Shapley value difference has the same classification success (accuracy 78.7%) of the list of 159 genes selected by CASh at *p *< 0.01. The same classification accuracy of 78.7% is also achieved by the list of genes obtained from the 160 ones with the highest Shapley value difference after that 47 genes with *p*-value from CASh greater than 0.01 are removed (Figure 5(c)).

**Figure 6:**
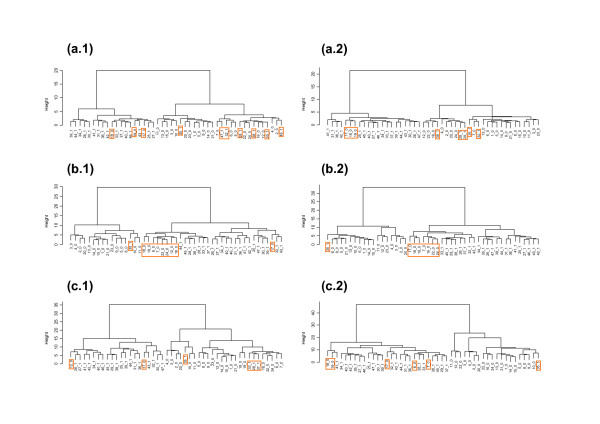
**Hierarchical clusterings on genes selected by CASh and t-test for same numbers of genes**. Hierarchical clustering (Ward method, Euclidean distance) of 47 subjects (columns): on 33 genes from CASh (a.1) and t-test (a.2); on 159 genes from CASh (b.1) and t-test (b.2); on 434 genes from CASh (c.1) and t-test (c.2). In subject labels, 1 means exposed subject, whereas 0 means non-exposed subject. Orange rectangles highlight misclassified subjects.

To compare the classification success of the lists of genes selected by CASh and t-test, we performed hierarchical clustering for equal numbers of genes (Figure 6). Table 3 shows a similar high level of classification accuracy for lists of genes by CASh and t-test, with a small increment in accuracy (4.2%) for t-test lists with 33 and 159 genes.

**Table 3:** Classification performance of lists of genes from t-test and CASh.

**Gene list size**	**CASh**	**t-test**
33	78.7%	82.9%
159	78.7%	82.9%
434	87.2%	87.2%

Classification acuracy based on clusters provided by hierarchical clustering performed on lists of genes selected by CASh and t-test with equal number of genes. Gene lists size is given by the number of genes selected by CASh at *p *smaller than 0.001, 0.01 and 0.05, respectively.

Over-representation of biological themes obtained by the pathway-finding tool DAVID [20] using the list of 434 genes with unadjusted *p*-values < 0.05 from CASh is presented in Table 4. Since the game theory method yielded 434 genes with *p *< 0.05 whereas the t-test yielded much more genes for the same level of *p *(see Table 1), we selected the same number of genes (with lowest *p*-values) from the t-test, to have equally sized gene lists for DAVID. The over-represented biological themes on the CASh list are compared with the over-represented biological themes in the list of 434 genes with smallest *p*-value in the t-test (Table 5).

**Table 4:** Over-represented annotation terms in genes selected by CASh.

**Functional Category**	**Term**	**efc**	***p*-value**
SP_PIR_KEYWORDS	Lectin	2,898	0,010
SP_PIR_KEYWORDS	elongation factor	5,367	0,011
SP_PIR_KEYWORDS	sh3 domain	2,402	0,014
SP_PIR_KEYWORDS	Symport	3,340	0,015
GOTERM_MF_ALL	translation elongation factor activity	4,579	0,019
GOTERM_MF_ALL	sugar binding	2,564	0,021
BIOCARTA	h_mapkPathway:MAPKinase Signaling Pathway	2,627	0,022
UP_SEQ_FEATURE	cross-link:Glycyl lysine isopeptide (Lys-Gly) (interchain with G-Cter in & ubiquitin)*	4,318	0,023
COG_KOG_ONTOLOGY	Inorganic ion transport and metabolism	3,378	0,023
UP_SEQ_FEATURE	repeat:Solcar 3	6,045	0,023
INTERPRO_NAME	IPR001452:Src homology-3	2,183	0,026
GOTERM_MF_ALL	FAD binding	5,698	0,027
SP_PIR_KEYWORDS	Disease mutation	9,661	0,032
UP_SEQ_FEATURE	repeat:Solcar 1	5,374	0,032
UP_SEQ_FEATURE	repeat:Solcar 2	5,374	0,032
SP_PIR_KEYWORDS	ubl conjugation	2,115	0,032
UP_SEQ_FEATURE	metal ion-binding site:Magnesium (via carbonyl oxygen)	9,068	0,036
INTERPRO_NAME	IPR001440:Tetratricopeptide TPR_1	2,478	0,038
INTERPRO_NAME	IPR013026:Tetratricopeptide region	2,478	0,038
SP_PIR_KEYWORDS	protease	1,668	0,038
GOTERM_BP_ALL	tRNA processing	3,707	0,040
SMART_NAME	SM00326:SH3	2,113	0,041
INTERPRO_NAME	IPR011990:Tetratricopeptide-like helical	2,116	0,043
GOTERM_MF_ALL	carbohydrate binding	2,1019	0,044
SP_PIR_KEYWORDS	Transferase	4,684	0,048
SP_PIR_KEYWORDS	Ubl conjugation*	4,684	0,048
BIOCARTA	h_gleevecpathway:Inhibition of Cellular Proliferation by Gleevec	4,446	0,051
UP_SEQ_FEATURE	domain:SH3*	2,303	0,053
KEGG_PATHWAY	HSA04540:GAP JUNCTION*	2,813	0,055
GOTERM_CC_ALL	obsolete cellular component*	4,403	0,056
SMART_NAME	SM00028:TPR	2,452	0,059
GOTERM_MF_ALL	purine nucleotide binding*	1,253	0,061
SMART_NAME	SM00177:ARF*	4,086	0,067
SP_PIR_KEYWORDS	inner membrane	2,373	0,069
GOTERM_CC_ALL	spindle pole	6,604	0,069
SP_PIR_KEYWORDS	transport	1,305	0,069
GOTERM_MF_ALL	aspartic-type endopeptidase activity	6,411	0,073
INTERPRO_NAME	IPR002067:Mitochondrial carrier protein	3,907	0,076
GOTERM_BP_ALL	biopolymer metabolism*	1,153	0,080
GOTERM_BP_ALL	secretion	1,719	0,084
BIOCARTA	h_erkPathway:Erk1/Erk2 Mapk Signaling pathway	3,613	0,086
BIOCARTA	h_fMLPpathway:fMLP induced chemokine gene expression in HMC-1 cells	3,613	0,086
GOTERM_MF_ALL	nucleotide binding*	1,201	0,087
SP_PIR_KEYWORDS	lipid transport	3,680	0,088
SP_PIR_KEYWORDS	nucleotide-binding	1,253	0,088
GOTERM_MF_ALL	microtubule binding	3,663	0,089
GOTERM_BP_ALL	macromolecule metabolism*	1,100	0,092
SP_PIR_KEYWORDS	endocytosis	2,800	0,097
GOTERM_BP_ALL	DNA metabolism	1,375	0,098
INTERPRO_NAME	IPR005123:2OG-Fe(II) oxygenase*	5,442	0,099

Overview of significant over-represented functional themes and pathways within the lists of most significant 434 genes selected by CASh. Only functional terms and pathways with *p*-value smaller than 0.1 are listed. The enrichment fold change (efc) is shown in the third column. Terms which are significantly over-represented also in genes from t-test (Table 5) are marked by *.

**Table 5:** Over-represented annotation terms and pathways in genes selected by t-test.

**Functional Category**	**Term**	**efc**	***p*-value**
UP_SEQ_FEATURE	cross-link:Glycyl lysine isopeptide (Lys-Gly) (interchain with G-Cter in & ubiquitin)	6,320	0,000
GOTERM_MF_ALL	guanyl nucleotide binding	2,344	0,000
GOTERM_MF_ALL	GTP binding	2,256	0,001
INTERPRO_NAME	IPR000217:Tubulin	6,384	0,001
INTERPRO_NAME	IPR002452:Alpha tubulin	9,120	0,001
INTERPRO_NAME	IPR008280:Tubulin/FtsZ, C-terminal	6,384	0,001
SP_PIR_KEYWORDS	Ubl conjugation	7,089	0,001
INTERPRO_NAME	IPR000558:Histone H2B	7,980	0,002
INTERPRO_NAME	IPR001660:Sterile alpha motif SAM	4,966	0,002
GOTERM_CC_ALL	Chromatin	2,683	0,003
SMART_NAME	SM00427:H2B	7,422	0,003
INTERPRO_NAME	IPR000047:Helix-turn-helix motif, lambda-like repressor	5,107	0,004
GOTERM_CC_ALL	Chromosome	2,139	0,005
GOTERM_BP_ALL	protein metabolism	1,243	0,005
SP_PIR_KEYWORDS	gtp-binding	2,122	0,007
SP_PIR_KEYWORDS	dna-binding	1,444	0,007
INTERPRO_NAME	IPR003008:Tubulin/FtsZ, GTPase	5,804	0,008
SMART_NAME	SM00454:SAM	4,453	0,008
PIR_SUPERFAMILY_NAME	SF002306:tubulin	8,354	0,009
UP_SEQ_FEATURE	nucleotide phosphate-binding region:GTP	2,107	0,013
KEGG_PATHWAY	HSA04540:GAP JUNCTION	3,353	0,014
SP_PIR_KEYWORDS	chromosomal protein	2,658	0,017
GOTERM_BP_ALL	microtubule-based movement	3,257	0,017
GOTERM_BP_ALL	macromolecule metabolism	1,154	0,019
GOTERM_BP_ALL	chromatin assembly or disassembly	2,412	0,020
GOTERM_BP_ALL	cytoskeleton-dependent intracellular transport	3,144	0,020
SP_PIR_KEYWORDS	DNA binding	1,786	0,021
GOTERM_MF_ALL	GTPase activity	2,252	0,021
UP_SEQ_FEATURE	DNA-binding region:Homeobox	3,447	0,025
GOTERM_BP_ALL	protein polymerization	3,398	0,027
UP_SEQ_FEATURE	domain:SAM	5,618	0,028
INTERPRO_NAME	IPR007124:Histone-fold/TFIID-TAF/NF-Y	2,883	0,030
GOTERM_MF_ALL	DNA binding	1,272	0,034
GOTERM_BP_ALL	glycoprotein biosynthesis	2,542	0,034
INTERPRO_NAME	IPR007125:Histone core	3,192	0,035
GOTERM_BP_ALL	tRNA metabolism	2,481	0,038
GOTERM_CC_ALL	nucleosome	2,719	0,039
GOTERM_BP_ALL	nucleosome assembly	2,682	0,041
GOTERM_CC_ALL	cell surface	3,048	0,042
GOTERM_BP_ALL	cellular macromolecule metabolism	1,176	0,043
UP_SEQ_FEATURE	domain:SH3	2,408	0,043
SP_PIR_KEYWORDS	nucleosome core	2,999	0,044
GOTERM_BP_ALL	primary metabolism	1,087	0,044
GOTERM_MF_ALL	binding	1,060	0,045
GOTERM_MF_ALL	sequence-specific DNA binding	1,739	0,046
GOTERM_BP_ALL	cellular protein metabolism	1,173	0,049
UP_SEQ_FEATURE	repeat:Spectrin 5	7,584	0,053
UP_SEQ_FEATURE	repeat:Spectrin 6	7,584	0,053
UP_SEQ_FEATURE	repeat:Spectrin 7	7,584	0,053
UP_SEQ_FEATURE	repeat:Spectrin 8	7,584	0,053
UP_SEQ_FEATURE	repeat:Spectrin 9	7,584	0,053
GOTERM_CC_ALL	obsolete cellular component	4,403	0,056
INTERPRO_NAME	IPR002017:Spectrin repeat	2,483	0,058
GOTERM_BP_ALL	anion transport	2,465	0,059
GOTERM_BP_ALL	metabolism	1,070	0,062
GOTERM_MF_ALL	purine nucleotide binding	1,255	0,062
GOTERM_BP_ALL	cellular metabolism	1,073	0,068
GOTERM_MF_ALL	transcription factor activity	1,398	0,068
GOTERM_MF_ALL	L-ascorbic acid binding	6,552	0,071
GOTERM_BP_ALL	regulation of mitosis	4,008	0,071
SP_PIR_KEYWORDS	vitamin c	6,499	0,072
SMART_NAME	SM00177:ARF	3,958	0,072
GOTERM_MF_ALL	carboxylic ester hydrolase activity	2,621	0,073
GOTERM_BP_ALL	chromatin assembly	2,338	0,073
INTERPRO_NAME	IPR006162:Phosphopantetheine attachment site	6,384	0,074
INTERPRO_NAME	IPR006620:Prolyl 4-hydroxylase, alpha subunit	6,384	0,074
GOTERM_CC_ALL	microtubule cytoskeleton	1,816	0,076
GOTERM BP ALL	microtubule-based process	2,127	0,077
GOTERM_MF_ALL	nucleotide binding	1,206	0,083
SMART_NAME	SM00702:P4Hc	5,938	0,084
GOTERM_MF_ALL	nucleic acid binding	1,150	0,087
INTERPRO_NAME	IPR001715:Calponin-like actin-binding	2,902	0,087
GOTERM_BP_ALL	biopolymer metabolism	1,150	0,090
GOTERM_MF_ALL	enzyme regulator activity	1,402	0,091
GOTERM_BP_ALL	glycoprotein metabolism	2,043	0,091
SMART_NAME	SM00033:CH	2,827	0,093
GOTERM_MF_ALL	enzyme activator activity	1,737	0,097
INTERPRO_NAME	IPR001526:CD59 antigen	5,472	0,098
INTERPRO_NAME	IPR005123:2OG-Fe(II) oxygenase	5,472	0,098
INTERPRO_NAME	IPR006703:AIG1	5,472	0,098

Overview of significant over-represented functional themes and pathways within the lists of most significant 434 genes selected by t-test. Only functional terms and pathways with *p*-value smaller than 0.1 are listed.

Upon t-test analysis DAVID returned more modified pathways (*n *= 84) than after CASh selection (*n *= 50). CASh-based pathway identification shares 11 annotation terms with t-test analysis-based pathway selection.

### Simulation study

In microarray studies, the detection of differential gene expression under two different conditions is very important. On the other hand, also the detection of differential gene expression variance may allow to identify experimental variables that affect different biological processes and accuracy of DNA microarray measurements. So, in this simulation we compare the performance of CASh and t-test in selecting genes which differ between two conditions in terms of average expression or expression variance.

**Figure 7:**
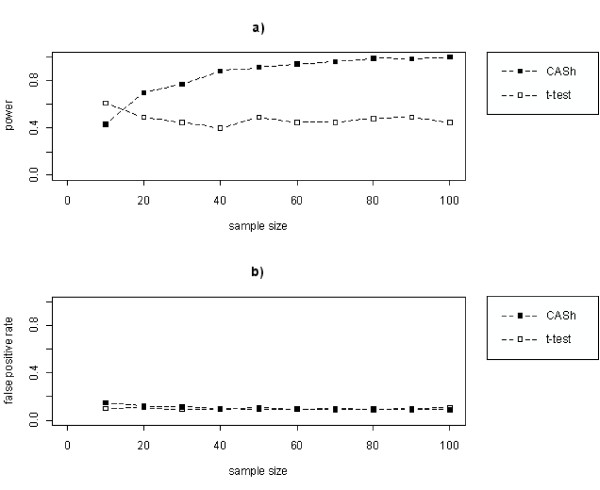
**Simulation results for CASh and t-test**. Estimated effect of sample size on the statistical power (left panel) and on the false positive rate (right panel)of CASh (lines with black squares) and the t-test (lines with white squares).

To assess the statistical power of CASh as a function of the sample size, we conducted a simulation study. We compared the performance of CASh against t-test on a simulated gene expression data-set of *n *= 1000 genes obtained by random samples from normal distributions under two simulated conditions: 90% of genes were sampled from the same distribution under the two conditions; the remaining 10% of genes was split in two groups of equal size: one group of genes 2-fold different in average expression between the two conditions and another group characterized by different variability across measures under the two conditions. Both CASh and t-test were applied on the simulated data-set, and genes with *p*-value smaller than predefined cutoffs, used to control the false positive rate at 0.1 in both methods, were selected. Figure 7 shows that the power of the t-test converges to 0.5 as expected, since half of the genes sampled from different distributions have a fold change not equal to 1. Differently, CASh converges to 1.

## Discussion

The purpose of our study is to introduce a new method (CASh) to analyze gene expression data, which combines the game theory notion of relevance index [6] with the notion of statistical significance. We illustrate the framework's utility by applying it to a published data-set [14] and the results of this application are discussed and compared with the output of a classical analysis for differential expression detection. A more detailed discussion on the statistical issues related to *p*-value generation in CASh is provided in Additional files [see Additional file 2].

Looking at the intersections of the set of genes selected by CASh with the set of genes selected by t-test, for each level of *p *in Table 1, we have that about 50% of genes selected by CASh are also selected by t-test. The different results obtained by CASh and t-test, are not very surprising. In fact, as further explained in *Methods*, the two approaches select relevant genes using different criteria. The t-test selects genes according to their individual differential expression between the two study conditions. Using the t-test, genes are considered significant on the basis of the difference of their expression profile between two conditions: gene *i *is called significant when its *p*-value is sufficiently small. The CASh method keeps into account the expression of each gene under two conditions, but the added value of the Shapley value is the ability to highlight the contribution of those genes which consistently interact with other genes. The CASh method calculates the relevance of genes as their average marginal contribution over all possible permutations of genes. Therefore, genes with the highest relevance are those that likely explain the difference between the two conditions, because they play an important role (on average) over all possible permutations, not only with respect to their individual differential expression.

Overlap rate of lists of genes generated according to different methods is shown in Figure 3. CASh method and differential Shapley value show a bigger overlap with the list provided by t-test than the list provided by fold-change. As far as we know, this is the first time that this result is reported on a real microarray data-set. Lists of genes selected from CASh and differential Shapley value show a large overlap, that for more then 50 selected genes varies between 70% and 80%.

The structure of the main representative groups provided by hierarchical clustering and *K*-means clustering based on the set of genes with differential expression and genes selected by CASh at < 0.01 shows gene expression profiles discriminating between the two areas.

In addition, hierarchical clustering and *K*-means clustering based on the set of genes selected by CASh, highlight a group of non-exposed subjects with homogenous levels of expression closer to another homogenous group of exposed subjects. To assess the reliability of this third cluster, we applied *K*-means clustering with *K *= 3 instead of *K *= 2, and we used the notion of mutual information in the framework of information theory [21]. Specifically, cluster-wise mutual information (CMI) relates the distributions of two random variables to each other providing a score which represents the amount of information that the distribution of one variable encodes about the other variable. CMI scores show that the reliability of the third cluster is very low in comparison with the two major clusters, suggesting that both lists of genes identified by CASh and t-test best classify subjects in only two groups (Table 6).

**Table 6:** Cluster-wise mutual information of *K*-means clustering.

**Procedure**	**clusters**	**size**	**CMI score**
CASh	cluster 1	28	0.2369
	cluster 2	18	0.2943
	cluster 3	1	0.0255

t-test	cluster 1	25	0.2825
	cluster 2	19	0.2922
	cluster 3	3	0.0733

Cluster-wise mutual information (CMI) scores computed on clusters of subjects as provided by *K*-means with *K *= 3 (genes selected by CASh and t-test at *p *< 0.01).

Table 3 shows that both CASh and t-test achieve a good separation performance when hierarchical clustering is applied to lists of genes of equal size. We are aware that clustering technique is not a classification procedure (see [22] for a comparison of different gene selection algorithm using performance on classification methods), but rather a method to reveal structural information in a data set. Therefore, achieving high levels of accuracy in clustering means that the information related to selected genes is sufficient to efficiently characterize the study conditions.

The same classification performance in terms of accuracy of clusters shown in Figures 5(b) and 5(c) suggests that genes with smallest *p*-value from CASh are the most informative among those with highest differential Shapley value. This fact may be explained by the ability of CASh method to provide genes with more stable Shapley value when small *p*-values are considered.

We also compared the medians of the sample statistics of the Shapley value of two genes, MFD1 and NFKBIA, with the same differential Shapley value but very different *p*-values from CASh. While the difference between TP and PR of the medians of Shapley value statistics for gene MFSD1 (*p *= 0.029) is zero, the corresponding difference of medians for the gene NFKBIA (*p *= 0.004) is larger than the differential Shapley value. On this particular instance, this result is consistent with the claimed ability of CASh to select genes with stable Shapley value. Note also that NFKBIA may be involved in diverse biological processes such as cell proliferation, differentiation, apoptosis and metastasis [23,24].

Among the genes selected by CASh only at *p *< 0.0001 (see Table 2), oligodendrocyte transcription factor 1 (OLIG1) was recently described in [25] as a prognostic marker for non-small cell lung cancer (NSCLC). Hormone-sensitive lipase (Lipe) is known to catalyze both the release of fatty acids from storage triglycerides in adipocytes and the liberation of cholesterol from cholesterol esters in steroidogenic tissues playing a key role in energy metabolism [26,27]. TMEPAI, an androgen induced gene, was found up-regulated in a time- and concentration-specific manner in prostate cancer cells (LNCaP) [28].

SRCRB4D contains 4 group B scavenger receptor cysteine-rich (SRCR) domains, a group of proteins known to be involved in the development of the immune system and the regulation of both innate and adaptive immune responses [29]. The sequence of human RBM6 is identical to DEF-3, that was found as differentially expressed during myelopoiesis [30], and of the lung cancer antigen NY-LU-12 [31].

For the seven genes selected by both CASh and t-test (*p *<0.0001) it is not clear at this moment exactly how they biologically relate to exposure to air pollution. We simply remark that DUSP15 encodes a protein that belongs to the protein-tyrosine phosphatase family, having both protein-tyrosine phosphatase activity and serine/threonine-specific phosphatase activity.

Given the properties of air pollutants in the TP region, one would hypothetically expect modifications of pathways related to (pre-)cancerous events and immune disorders. CASh-based pathway identification shares 11 common annotation terms with t-test analysis-based pathway selection (see Table 4); none of these however show any known biological annotation referring to carcinogenis or immunotoxicity. Of more interest are the differences between these two selections: t-test-based analysis demonstrates pathways related to nucleosome function and microtubule structure and function which may be associated with observed differences in genotoxic damage between children from TP and from PR [14], while CASh retrieves affected MAPK-signaling pathways which may refer to deregulation of cellular growth predisposing to tumorigenesis [32].

## Conclusion

In this paper, a new method to analyze the relevance of genes under a given condition is studied. CASh integrates the game theory model introduced in [6] with a novel Boostrap-based test procedure that allows to compare a gene relevance index computed within game theory, i.e., the Shapley value, which reflects the joint expression behavior of genes. We argued that the added value of CASh with respect to the approach in [6] is to perform statistical inference based on the distributions of the sample statistics of microarray games and the corresponding statistics of Shapley values.

On simulated data where differential expression and differential variability of genes characterize two conditions, we showed that CASh affords more power than the t-test at the same false positive rate. CASh and t-test were applied to data published in [14], concerning the gene expression measured in children from the Czech Republic differentially exposed to air pollution. A group of children lived in the area of TP, which is characterized by relatively high levels of air pollution and the other in the less polluted area of PR. Hierarchical clustering and K-means clustering are used to group together individuals on the basis of the expression patterns of genes selected by CASh and t-test, and to compare the performance of the two methods in selecting genes that jointly act in characterizing samples from the polluted and the non-polluted areas. Clustering methods show that the lists of genes provided by CASh and t-test are informative enough to discriminate between TP subjects and PR subjects on the basis of their gene expression profiles.

Most of genes selected by CASh at *p *< 0.0001 are involved in important processes related to the mechanism of carcinogenesis. While most of the gene categories shown in Tables 3 and 4 cannot yet be toxicologically interpreted, it is demonstrated that t-test analysis generates presumably relevant pathways, e.g. related to nucleosome and microtubuli function, but also misses a few, e.g. related to cell signaling and growth regulation, which are retrieved by CASh. At the level of identified pathways as affected by exposure to air pollution in Teplice children, it is the combination of both methods that yields most of the relevant information regarding genes with a potential impact in regulation of complex pathways predisposing to tumorigenesis. It is therefore recommended to apply CASh and parametric tests for differential expression in combination.

## Methods

### Game Theory

In this section we introduce some basic game theoretical notions and definitions. A *coalitional game *is a pair (*N*, *v*), where *N *denotes a finite set of *players *and *v *: 2^*N *^→ ℝ the *characteristic function*, with *v*(∅) = 0. Often we identify a coalitional game (*N*, *v*) with the corresponding characteristic function *v*. A group of players *T *⊆ *N *is called a *coalition *and *v*(*T*) is the *worth *of the coalition *T*.

The *unanimity game *(*N*, *u*_*R*_) based on *R *⊆ *N *is the game described by *u*_*R*_(*T*) = 1 if *R *⊆ *T *and *u*_*R*_(*T*) = 0, otherwise. Every coalitional game (*N*, *v*) can be written as a linear combination of unanimity games in a unique way, i.e. *v *= ∑_*S*⊆*N*, *S*≠∅ _*λ*_*S *_(*v*) *u*_*S *_(see for instance [33]. The coefficients ${\left(\lambda_{S}(v)\right)}_{S\in 2^{N}\backslash \{\emptyset \}}$ are called *unanimity coefficients *or *dividends *of the game (*N*, *v*).

Given a coalitional game (*N*, *v*), an *allocation *or *payoff vector *is a vector (*x*_*i*_)_*i*∈*N *_∈ ℝ^*N *^assigning to player *i *∈ *N *the amount *x*_*i*_.

A *solution *for a class of coalitional games is a function *ψ *that assigns a payoff vector *ψ*(*v*) to every coalitional game in the class. A well-known solution for coalitional games is the *Shapley value*, introduced by [7].

The Shapley value assigns to each player his average marginal contribution over all the possible orderings, i.e. permutations, of the players. Formally, given a coalitional game (*N*, *v*), the Shapley value assigns to player *i *∈ *N*:

(1) $$\phi_{i}(v)=\frac{1}{n!}\sum_{\pi }\left(v(P(\pi ;i)\cup \{i\})-v(P(\pi ;i)\right)$$

where *π *is a permutation of the players, *P*(*π*; *i*) is the set of players that precede player *i *in the permutation *π *and *n *is the cardinality of *N*.

In [6], the definition of *microarray game *was introduced as a coalitional game (*N*, $\bar{v}$) with the objective to stress the relevance ('sufficiency') of groups of genes in relation to a specific condition. Let *N *= {1,...,*n*} be a set of genes. On a single microarray experiment on *N*, a sufficient requirement to realize in a coalition *S *⊆ *N *the association between a condition and an expression property is that all the genes showing that expression property belong to coalition *S *(*sufficiency principle*). Different expression properties for genes might be considered like, e.g., under- or over-expression, strong variation, abnormal expression etc. A group of genes *S *⊆ *N *which realizes the association between the expression property and the condition on a single array is called a *winning coalition *for that array. For example, consider a single microarray experiment on a set of genes *N *= {1, 2,...,10} under a given condition (e.g., exposure to air pollution) and suppose that only genes 1, 3 and 7 show the expression property (e.g., over-expression). Then, each set of genes *S *⊆ *N *with 1, 3, 7 ∈ *S *is a winning coalition in such an experiment.

Moving to *k *≥ 1 microarray experiments on *N*, we refer to a Boolean matrix **B **∈ {0,1}^*n *× *k*^, where the Boolean values 0 – 1 represent two complementary expression properties, for example the property of normal expression (coded by 0) and the property of abnormal expression (coded by 1). Let **B**._*j *_be the *j*-th column of **B**. We define the *support *of **B**._*j*_, denoted by *sp*(**B**._*j*_), as the set *sp*(**B**._*j*_) = {*i *∈ *N *| **B**_*ij *_= 1}.

The *microarray game *corresponding to **B **is the coalitional game (*N*, $\bar{v}$) with *N *= {1,...,*n*} and where $\bar{v}$: 2^*N *^→ ℝ_+ _is such that $\bar{v}$(*T*) is the rate of occurrences of the coalition *T *as a winning coalition, i.e., is the rate of occurrences of the coalition *T *as a superset of the supports in the Boolean matrix **B**. in formula, we define $\bar{v}$(*T*), for each *T *∈ 2^*N *^\ {∅}, as the value

(2) $$\bar{v}(T)=\frac{card(\Theta (T))}{k}$$

where Θ(*T*) = {*j *∈ *K *| *sp*(**B**._*j*_) ⊆ *T*, *sp*(**B**._*j*_) ≠ ∅}, with *K *= {1,...,*k*} and where *card*(Θ(*T*)) is the cardinality of Θ(*T*). Finally, we define $\bar{v}$(∅) = 0.

Note that the expression of a gene is a continuous variable which hypothetically may assume whatever value across different samples, then it is not at all easy to identify good criteria to discriminate between different expression properties. The binarization method used in this work is described in section *Data processing for CASh*. For alternative binarization methods in gene expression analysis, see for instance [34,35].

### Comparative Analysis of Shapley Value

Consider two groups of microarray experiments on the same set of genes *N *= {1,...,*n*}, respectively collected under two different conditions 1 and 2. Let **B**^1 ^∈ {0, 1}^*n *× *k *^and **B**^1 ^∈ {0, 1}^*n *× *h *^be two Boolean matrices, where **B**^1 ^is obtained via a discretization procedure from an expression data set with *k *biological samples under condition 1, and **B**^2 ^is obtained via a discretization procedure from an expression data set with *h *biological samples under condition 2.

Let ${\bar{v}}^{1}$, ${\bar{v}}^{2}$ be the microarray games corresponding to the Boolean matrices **B**^1 ^and **B**^2^, respectively. Let *ϕ*(${\bar{v}}^{1}$) be the Shapley value on the game ${\bar{v}}^{1}$ and let *ϕ*(${\bar{v}}^{2}$) be the Shapley value on the game ${\bar{v}}^{2}$.

Consider the following absolute difference of Shapley values

(3) $$\delta_{i}(\phi ({\bar{v}}^{1}),\phi ({\bar{v}}_{2})):=|\phi_{i}({\bar{v}}^{1})-\phi_{i}({\bar{v}}^{2})|,$$

for each *i *∈ *N*, where *ϕ*_*i*_(${\bar{v}}^{1}$) is the Shapley value of gene *i *in the microarray game corresponding to the Boolean matrix **B**^1 ^and *ϕ*_*i*_(${\bar{v}}^{2}$) is the Shapley value of gene *i *in the microarray game corresponding to the Boolean matrix **B**^2^.

We formally present a procedure (Algorithm 1) to test the null hypothesis that each gene in *N *has the same Shapley value between the two conditions 1 and 2. In fact we want to test the *null hypothesis δ*_*i*_(*ϕ*(${\bar{v}}^{1}$), *ϕ*(${\bar{v}}^{2}$)) = 0 against the *alternative hypothesis δ*_*i*_(*ϕ*(${\bar{v}}^{1}$), *ϕ*(${\bar{v}}^{2}$)) ≠ 0. More precisely, we introduce a test procedure based on a non-parametric Bootstrap method of re-sampling with replacement (see [12,13] as general introduction to Bootstrap methods; see [36] as a Bootstrap application to microarray analysis), which is able to test the null hypothesis of no difference between the means of two random samples without assuming under the null hypothesis that the probability distributions in the populations are the same.

### Algorithm 1

INPUT:

- Two Boolean matrices **B**^1 ^∈ {0,1}^*n *× *k*^, **B**^2 ^∈ {0, 1}^^*n *× *h*^^, with *n*, *k*, *h *∈ {1, 2,...};

- an integer number *b *of Bootstrap re-samples (with replacement).

OUTPUT:

- a Bootstrap estimation of the null distribution of Shapley value differences on the *n *genes;

- a vector of *n *(un-adjusted for multiple comparisons) estimated *p*-values.

BEGIN:

- Compute the *observed Shapley value difference *$\delta_{i}(\phi ({\bar{v}}^{1}),\phi ({\bar{v}}^{2}))=|\phi_{i}({\bar{v}}^{1})-\phi_{i}({\bar{v}}^{2})|$ for each *i *∈ *N*, where ${\bar{v}}^{1}$ and ${\bar{v}}^{2}$ are the microarray game correspondingto **B**^1 ^and **B**^2^, respectively.

FOR *r *: 1 TO *b *BEGIN:

- Compute the *r*^1^-th Bootstrap re-sample (with replacement) on the column indices {1,...,*k*} of **B**^1^; compute the *r*^2^-th Bootstrap re-sample (with replacement) on the column indices {1,...,*h*} of **B**^2^, respectively.

- Define the Boolean matrix $B^{s^{r,1}}\in {\left\{0,1\right\}}^{n\times k}$ corresponding to the *r*^1^-th re-sample and the Boolean matrix $B^{s^{r,2}}\in {\left\{0,1\right\}}^{n\times h}$ corresponding to the *r*^2^-th re-sample.

- Compute the *Bootstrap Shapley value difference *$\beta_{i}^{r}(\phi ({\bar{v}}_{r}^{1}),\phi ({\bar{v}}_{r}^{2}))=|(\phi_{i}({\bar{v}}_{r}^{1})-\phi_{i}({\bar{v}}^{1}))-(\phi_{i}({\bar{v}}_{r}^{2})-\phi_{i}({\bar{v}}^{2}))|$, for each *i *∈ *N*, where ${\bar{v}}_{r}^{1}$, ${\bar{v}}_{r}^{2}$ are the microarray games corresponding to the Boolean matrices $B^{s^{r,1}}$ and $B^{s^{r,2}}$, respectively.

END.

- for each *i *∈ *N*, compute the (un-adjusted for multiple comparisons) estimate Achieved Significance Level (ASL) or *p*-value *p*_*i *_of each gene *i *∈ *N *as follows $p_{i}=\frac{card\left(\left\{r:\beta_{i}^{r}(\phi ({\bar{v}}_{r}^{1}),\phi ({\bar{v}}_{r}^{2}))\geq \delta_{i}(\phi ({\bar{v}}^{1}),\phi ({\bar{v}}^{2}))\right\}\right)}{b}$.

END.

In Additional files, a more detailed version of the pseudo-code of Algorithm 1 [see Additional file 1] and its implementation [see Additional file 4] are given. A discussion on the generation of raw *p*-values using bootstrap method and the related procedures to adjust *p*-values for multiple hypothesis testing is provided [see Additional file 2]. Calculations of CASh on a numerical instance are also illustrated [see Additional file 3].

### Description of data processing

We analyzed the microarray gene expression data published in [14]. Study subjects were children from the Teplice (TP) area in the north and from the rural Prachatice (PR) in the south of the Czech Republic, for a total of 47 children; 23 from the TP area and 24 from the PR area. For details on study population, collection and processing of blood, RNA isolation and microarray analysis of gene expression see [14].

#### Data pre-processing

Raw data files from ImaGene (BioDiscovery, Marina del Rey, CA, USA) published in [14] were uploaded into Expressionist Refiner Array (Genedata AG, Basel, Switzerland) for data transformation. Data transformations were applied in the following order: background was corrected according to [37,38]; LOWESS correction with a smoothing factor of 0.1 to remove any nonlinearity between the two channels was applied [39]; expression ratios of the subjects's sample with respect to the common reference sample were calculated using a specific bayesian algorithm to estimate the most likely expression signal given the measurements for the spot and background intensities. The following data were derived:

• Expression ratio = signal Cy5/signal Cy3;

• Signal to noise (S/N) ratio for each channel = $\frac{\text{signal}}{\text{background}}$;

• Relative error computed = $\frac{\text{signal Stdev}}{\text{signal}\times {\left(\text{Spot Area}\right)}^{\frac{1}{2}}}$.

For the analysis of the data the quality thresholds were set as follows:

• Relative error < 0.5;

• S/N ratio > 2.0;

• Saturated features masked.

In addition, only the transcripts were used which have, after the previously described filtering, at least 50% valid values per group, i.e. ≥ 12 valid values for the PR group and also ≥ 12 for the TP group. From the about 20000 spots on the microarray, 5873 fulfill the above described quality and filtering criteria and were used for further statistical analysis. These spots from the series of 47 experiments are represented as a gene expression matrix **X**, with *n *= 5873 (after filtering) rows and 47 columns, where the *i*-th row consists of a 47-elements expression vector **X**_*i*_. = (**X**_*i*1_,...,**X**_*i*47_), for a single gene sequence *i*.

On such a matrix, a t-test analysis was used to identify genes significantly differing in expression between the two groups of individuals (TP compared to PR).

### Data processing for CASh

The final matrix **X **of 5873 genes and 47 samples that was distilled from the data filtering and preparation as described above, was split in two distinct expression matrices, **X**^*TP *^and **X**^*PR*^, whose columns were selected from **X **accordingly to the 23 subjects from TP area and the 24 subjects from PR area.

First, a procedure aimed to discriminate over-regulated levels of gene expression with respect to expressions measured in the PR area was applied. Each continuous value in the vector **X**_*i*. _= (**X**_*i*1_,...,**X**_*i*47_) which was equal to or greater than *Mean *[$X_{i.}^{PR}$] + *Stdev*[$X_{i.}^{PR}$] was coded as 1, or as 0 if otherwise. Consequently, a Boolean matrix **B**^+ ^with n rows and 47 columns and with values {0, 1} was generated from **X**. Separately, a procedure aimed to discriminate under-regulated levels of gene expression with respect to expressions measured in the PR area was applied. Each continuous value in the vector **X**_*i*_. = (**X**_*i*1_,...,**X**_*i*47_) which was equal to or smaller than *Mean*[$X_{i.}^{PR}$] - *Stdev*[$X_{i.}^{PR}$] was coded as 1, or as 0 if otherwise. Consequently, a Boolean matrix **B**^- ^with n rows and 47 columns with values {0, 1} was also generated from **X**. According to the distinction between PR and TP biological samples, the Boolean matrix **B**^+ ^was split in two different Boolean matrices **B**^*TP*+ ^and **B**^*PR*+^, and the Boolean matrix **B**^- ^was split in two other Boolean matrices **B**^*TP*- ^and **B**^*PR*-^. By relation (2), from the Boolean matrix **B**^*TP*+ ^the microarray game ${\bar{v}}^{TP+}$ is defined and, in a similar way, the microarray game ${\bar{v}}^{TP-}$ from the Boolean matrix **B**^*TP*- ^is also defined.

In order to remove those genes whose high level of Shapley value may be attributed to chance, we applied the Bootstrap-based Algorithm 1. In practice, we applied Algorithm 1 (*b *= 1000) with **B**^*TP*+ ^in the role of **B**^1 ^and **B**^*PR*+ ^in the role of **B**^2 ^and the un-adjusted *p*-values were computed. In a similar manner, we applied Algorithm 1 (*b *= 1000) with **B**^*TP*- ^in the role of **B**^1 ^and **B**^*PR*- ^in the role of **B**^2 ^and the corresponding un-adjusted *p*-values were computed.

As further criterium for filtering, genes with Shapley value smaller than the mean plus the standard deviation in both microarray games ${\bar{v}}^{TP+}$ and ${\bar{v}}^{TP-}$ were filtered out. Following this criterium, 838 genes were selected in game ${\bar{v}}^{TP+}$ and 889 genes were selected in game ${\bar{v}}^{TP-}$) (see the highlighted intervals of Figure 1).

The overlap between two lists with the same number *n *of genes is defined as the following fraction

$$\text{overlap}(\%)=\frac{\text{number of genes in common between the two list}}{n}\times 100,$$

for *n *≥ 1.

#### Hierarchical cluster analysis and gene ontology

We used hierarchical clustering and K-means clustering to detect similarity relationships in gene expressions between TP and PR areas, based on the set of genes selected by CASh and t-test. In hierarchical clustering, all agglomerative hierarchical clusters were computed using the Euclidean distance between single vectors and the Ward method [40]. In K-means clustering, the algorithm of Hartigan and Wong [41] is used, the number of clusters a priori specified is *K *= 2 and the maximum number of iterations allowed is 10000. Before clustering analysis, we imputed missing values by the k-Nearest Neighbors method (*k *= 5) [42]. Heat-maps were representative of logged gene expression values, which are centered and scaled in the row direction. Statistical analysis were performed with Expressionist Pro from Genedata or the software R [see ]. Classification accuracy of clusters is measured as the percentage of correctly classified subjects. More precisely, classification accuracy was computed in two steps: first, each cluster is assigned to the area (TP or PR) with the majority of subjects in the cluster; second, the accuracy is computed according to the following ratio:

$$\text{accuracy}=\frac{\text{number of TP subjects in the cluster assigned to TP}+\text{number of PR subjects in the cluster assigned to PR}}{\text{total number of subjects}(=47)}.$$

For functional annotation analysis, we used the online software DAVID [20], which employs a modified Fishers exact test [43,44] to derive biological themes within particular gene sets defined by functional annotation. In this way, over-representation of a particular annotation term corresponding to a group of genes was quantified in terms of the *p*-value computed in the test procedure.

### Simulation study

A simulated gene expression data-set of of *n *= 1000 genes obtained by random samples from normal distributions under two simulated conditions which are denoted by a class variable *y *∈ {1, 2}. Nine hundred genes were randomly sampled from a normal distribution with mean= 1 and stdev = 1 under both conditions 1 and 2. The remaining 100 genes were slpit in two sets of target genes (i.e., to be discovered genes), since the parameters of the normal distributions from which the expression values are sampled change with the conditions: 50 genes were randomly sampled from a normal distribution with mean = 2 and stdev = 1 under condition 1, and from a normal distribution with mean = 1 and stdev = 1 under condition 2; remaining 50 genes were randomly sampled from a normal distribution with mean = 1 and stdev = 1 under condition 1, and from a normal distribution with mean = 1 and stdev = 2 under condition 2. To be processed by CASh, each randomly sampled continuous value of gene *i*, *i *= 1,..., 1000, under condition 1 (condition 2) which was equal to or greater than the average expression of gene *i *plus its standard deviation under condition 2 (condition 1) was coded as 1, or as 0 if otherwise.

## Authors' contributions

SM conceived the initial idea of Algorithm 1, supervised the application of CASh, developed *R *scripts for data processing and simulations and prepared the manuscript. DL and HG supervised the analysis for the identification of differentially expressed genes and over-represented biological terms. HG also performed data pre-processing, filtering and normalization. SB contributed to the supervision of the CASh statistical analysis. FM and JK conceived the idea of comparing CASh and parametric statistical analysis. JK, DL and JvD contributed to the biological interpretation of the results. FP contributed to the supervision of the game theory approach. All authors contributed to the discussion of the results and approved the final version of the manuscript.

## Supplementary Material

Additional file 1 **CASh pseudo-code and implementation**. contains details on the computation of Algorithm 1 and how this algorithm has been implemented in R language. Click here for file

Additional file 2 **Bootstrapping *p*-value estimations**. contains a discussion on the *p*-value estimation and how to deal with multiple comparison hypothesis in CASh. Click here for file

Additional file 3 **Example of calculations for CASh**. contains an example of calculations for CASh on a toy expression matrix. Click here for file

Additional file 4 **R code**. contains an R function to compute the Shapley value of microarray games. Click here for file
