# Supplementary material for: Combining Shapley value and statistics to the analysis of gene expression data in children exposed to air pollution
Source: BMC Bioinformatics. 2008 Sep 2;9:361. doi: 10.1186/1471-2105-9-361 (PMC2556684; doi:10.1186/1471-2105-9-361)
Supplement: Additional file 1 — CASh pseudo-code and implementation. contains details on the computation of Algorithm 1 and how this algorithm has been implemented in R language. [file 1471-2105-9-361-S1.pdf]

CASh pseudo-code and implementation

In this section we give a more detailed description of Algorithm 1. In addition, we show how this algorithm has been implemented.

We start from an alternative representation of the Shapley value, that can be given in terms of the unanimity coefficients $(\lambda_S(v))_{S\in 2^N\setminus\{\emptyset\}}$, that is:

$$\phi_i(v) = \sum_{S\subseteq N: i\in S} \frac{\lambda_S(v)}{s} \tag{1}$$

for each $i \in N$, where s is the cardinality of S.

In Methods we introduced the definition of microarray game corresponding to a given Boolean matrix. An equivalent way to calculate the microarray game $\bar{v}$ corresponding to a Boolean matrix $\mathbf{B}$ is as a sum of unanimity games as follows

$$\bar{v} = \frac{1}{k} \sum_{j\in K: sp(\mathbf{B}_{\cdot j})\neq\emptyset} u_{sp(\mathbf{B}_{\cdot j})}, \tag{2}$$

where $u_{sp(\mathbf{B}_{\cdot j})}$ is the unanimity game on $sp(\mathbf{B}_{\cdot j}) \subseteq N$, for each $j \in K$.

Note that the computation of the Shapley value $\phi(\bar{v})$ in a microarray game $\bar{v}$, in virtue of equations (1) and (2), is very easy, independently of the number of players in N. More precisely, we have that

$$\phi_i(\bar{v}) = \frac{1}{k} \sum_{j=1}^{k} \mathbf{R}_{ij} \tag{3}$$

for each $i \in N$, where the matrix $\mathbf{R} \in [0,1]^{n\times k}$ is such that for each $i \in N$ and each $j \in K$

$$\mathbf{R}_{ij} = \begin{cases} 0 & \text{if } \mathbf{B}_{ij} = 0 \\ \frac{1}{card(sp(\mathbf{B}_{\cdot j}))} & \text{otherwise} \end{cases} \tag{4}$$

and $card(sp(\mathbf{B}_{\cdot j}))$ is the cardinality of $sp(\mathbf{B}_{\cdot j})$.

We describe the nonparametric Bootstrap approach to estimate the (unadjusted for multiple comparisons) p-values in the next algorithm:

Algorithm 1

INPUT:

- Two Boolean matrices $\mathbf{B}^1 \in \{0,1\}^{n\times k}$, $\mathbf{B}^2 \in \{0,1\}^{n\times h}$, with $n, k, h \in \{1, 2, \ldots\}$;
- an integer number b of Bootstrap re-samples (with replacement).

OUTPUT:

- a Bootstrap estimation of the null distribution of Shapley value differences on the n genes;
- a vector of n (un-adjusted for multiple comparisons) estimated p-values.

Begin:

step 1 : Compute the matrix $\mathbf{R}^1 \in [0,1]^{n\times k}$according to relation (4) applied to Boolean matrix $\mathbf{B}^1$;
compute the matrix $\mathbf{R}^2 \in [0,1]^{n\times h}$according to relation (4) applied to Boolean matrix $\mathbf{B}^2$.

step 2 : Compute the *observed Shapley value difference* $\delta_i(\phi(\bar{v}^1),\phi(\bar{v}^2)) = \left|\frac{1}{k}\sum_{j=1}^{k}\mathbf{R}^1_{ij} - \frac{1}{k}\sum_{j=1}^{h}\mathbf{R}^2_{ij}\right|$ for each $i \in N$;

step 3 : for $r : 1$ to b $\Big\{$

step 4.r Let $\mathbf{s}^{r,1} = (s^{r,1}_j)_{j\in\{1,\dots,k\}} \in \{1,\dots,k\}^k$ and $\mathbf{s}^{r,2} = (s^{r,2}_j)_{j\in\{1,\dots,h\}} \in \{1,\dots,h\}^h$ be the vectors representing the r-th Bootstrap re-sample (with replacement) on the indeces $\{1,\dots,k\}$ and $\{1,\dots,h\}$, respectively.

step 5.r Consider the new Boolean matrix $\mathbf{B}^{\mathbf{s}^{r,1}} \in \{0,1\}^{n\times k}$ such that $\mathbf{B}^{\mathbf{s}^{r,1}}_j = \mathbf{B}^1_{s^{r,1}_j}$ for each $j \in \{1,\dots,k\}$ and the Boolean matrix $\mathbf{B}^{\mathbf{s}^{r,2}} \in \{0,1\}^{n\times h}$ such that $\mathbf{B}^{\mathbf{s}^{r,2}}_j = \mathbf{B}^2_{s^{r,2}_j}$ for each $j \in \{1,\dots,h\}$.

step 6.r Compute the *Bootstrap Shapley value difference*

$$\begin{aligned}&\beta^r_i(\phi(\bar{v}^1_r),\phi(\bar{v}^2_r)) :=\\ &:= \left|\big(\phi_i(\bar{v}^1_r)-\phi_i(\bar{v}^1)\big)-\big(\phi_i(\bar{v}^2_r)-\phi_i(\bar{v}^2)\big)\right|,\end{aligned} \tag{5}$$

for each $i \in N$, where $\bar{v}^1_r, \bar{v}^2_r$ are the microarray games corresponding to the Boolean matrix $\mathbf{B}^{\mathbf{s}^{r,1}}$ and $\mathbf{B}^{\mathbf{s}^{r,2}}$, respectively.

$\Big\}$

step 7 : for each $i \in N$, compute the (un-adjusted for multiple comparisons) estimate Achieved Significance Level (ASL) or p-value p_i of each gene $i \in N$ as follows

$$p_i = \frac{card\big(\{r : \beta^r_i(\phi(\bar{v}^1_r),\phi(\bar{v}^2_r))) \geq \delta_i(\phi(\bar{v}^1),\phi(\bar{v}^2))\}\big)}{b}. \tag{6}$$

End.

Remark 1 In order to preserve the ties among genes in each sample, on step 5.r, the entire columns of the Boolean matrices $\mathbf{B}^1$ and $\mathbf{B}^2$ are re-sampled according to the vectors $\mathbf{s}^{r,1}$ and $\mathbf{s}^{r,2}$ defined on step 4.r, for each $r \in \{1,\dots,b\}$.

Remark 2 Subtracting $\phi(\bar{v}^1_r)$ and $\phi(\bar{v}^2_r)$ in (5) from the Bootstrap Shapley values in the game defined on matrix $\mathbf{B}^{\mathbf{s}^{r,1}}$ and $\mathbf{B}^{\mathbf{s}^{r,2}}$, respectively, makes the Bootstrap Shapley values correspond to the null hypotheses $\delta_i(\phi(\bar{v}^1),\phi(\bar{v}^2))$ $= 0$.

Implementation of Algorithm 1

Algorithm 1 was implemented using the following R (www.r-project.org) code:

> $\mathbf{R}^1$ <- shapleymat($\mathbf{B}^1$)

> $\mathbf{R}^2$ <- shapleymat($\mathbf{B}^2$)

> $\mathbf{R}$ <- matrix(NA, n, (k+h))

> $\mathbf{R}$[,1:k] <- $\mathbf{R}^1$

> $\mathbf{R}$[,(k+1):(k+h)] <- $\mathbf{R}^2$

> *classes* <- c(array(1, dim=c(k)), array(2, dim=c(h)))

> library(multtest)

> OUTPUT <- MTP(X=$\mathbf{R}$, Y=*classes*, standardize=FALSE, B=b)

Where *shapleymat()* is the R function provided as additional file, MTP is function to perform resampling-based multiple hypothesis testing of the Bioconductur (www.bioconductor.org) package *multtest*. In arguments of MTP function, *classes* is a vector of class labels for column of matrix $\mathbf{B}$, representing the two conditions 1 and 2 and b is the number of Bootstrap re-samples. In order to see the Bootstrap estimation of the null distribution of Shapley value differences for all of the n genes type
> OUTPUT@rawp
In order to see the vector of n (un-adjusted for multiple comparisons) estimated p-values type
> OUTPUT@statistic
