# Supplementary material for: Combining Shapley value and statistics to the analysis of gene expression data in children exposed to air pollution
Source: BMC Bioinformatics. 2008 Sep 2;9:361. doi: 10.1186/1471-2105-9-361 (PMC2556684; doi:10.1186/1471-2105-9-361)
Supplement: Additional file 2 — Bootstrapping p-value estimations. contains a discussion on the p-value estimation and how to deal with multiple comparison hypothesis in CASh. [file 1471-2105-9-361-S2.pdf]

Bootstrapping p-value estimations

In microarray studies it is common that the the sample size is small and that the distribution of expression values differs from normality. In this situations, permutation and bootstrap tests may be appropriate for the identification of differentially expressed genes.

Following the bootstrap approach of Algorithm 1, the un-adjusted for multiple comparison p-values for each gene i is estimated as the proportion of permutation-based Shapley value differences $\delta_i^r(\phi(\bar{v}_r^1), \phi(\bar{v}_r^2))$ that are greater than the observed Shapley value difference $\delta_i(\phi(\bar{v}^1), \phi(\bar{v}^2))$. The estimated p-values provided by bootstrap methods (with replacement) are less exact than p-values obtained from permutation tests (without replacement) (see e.g. Dudoit et al.(2002, 2003)) but, as we already mentioned, can be used to test the null hypothesis of no differences between the means of two statistics (Efron and Tibshirani (1993)) without assuming that the distributions are otherwise equal (see also Bickel (2002)).

Following the approach in Storey and Tibshirani (2003), Figure 1 shows a density histogram of the of 5873 estimated p-values provided by Algorithm 1 on the data-set of 47 children in TP and PR, when $\bar{v}^{TP+}$ vs. $\bar{v}^{PR+}$ is considered. The dashed line is the density we would expect if all genes were null (i.e., with Shapley value not different between the two conditions TP and PR). The density histogram of p-values beyond 0.3 looks fairly flat, which indicates there are mostly null p-values in this region. According to Storey and Tibshirani (2003), the height of this flat proportion actually gives a conservative estimate of the overall proportion of null p-values (77.9%). For comparison we show in Figure 2 a density histogram of the of 5873 estimated p-values provided t-test. Here the region beyond 0.4 looks fairly flat and a conservative estimate of the overall proportion of null p-values is 68.5%.

Applying the Algorithm 1 to microarray data, thousands of null hypothesis can be tested separately; so we would need to consider the problem of multiple comparison. In fact, if n is the number of statistical tests, each performed at level α, if the tests are independent, the expected number of false positive is αn, which is very large for large n.

It is possible to alleviate this problem by adjusting the individual p-value of the tests for multiplicity. Several methods have been proposed in literature to tackle this problem (see for a summary Amaratunga and Cabrera (2004)), mainly assuming independence of the test statistics. In Algorithm 1, test statistics are likely not independent; in fact they are statistics on the Shapley value distribution in the population of genes, which should be representative of the relevance of each gene (interacting with many others) in determining the association between the genes expression properties of groups of genes

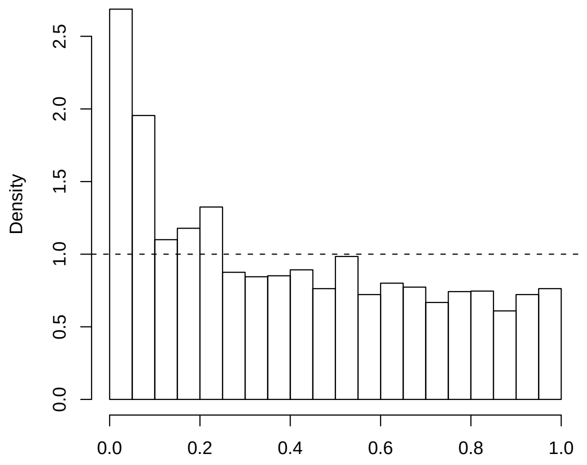

Figure 1: density histogram of the of estimated p-values provided by Algorithm 1.

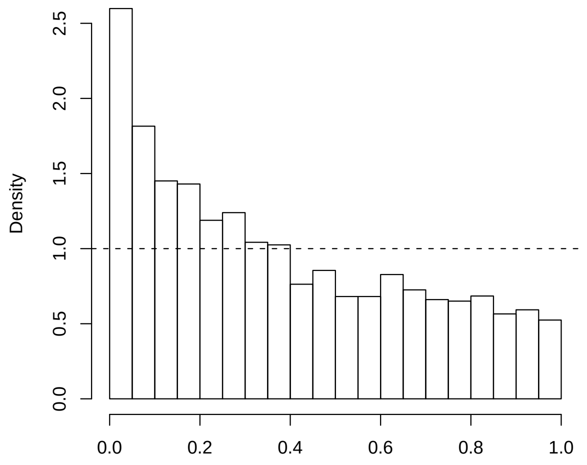

Figure 2: density histogram of the of p-values provided by t-test.

and the study conditions. On the other hand, the problem of multiplicity is still there, but to establish its entity is even harder with respect to the case of test statistics independency.

Moreover, given the very high number of null hypothesis tested in a typical microarray game, aggressively adjusting the p-values for multiplicity could seriously impede the ability of the test to find genes with respective relevance index which are truly different under the two biological conditions at hand.

Traditional statistical procedures often control the family-wise error rate (FWER), i.e. the probability that at least one of the true null hypothesis is rejected. Classical p-value adjustment methods for multiple comparisons which control FWER have been found to be too conservative in analyzing differential expression in large-screening microarray data, and the False Discovery Rate (FDR), i.e. the expected proportion of false positives among all positives, has been recently suggested as an alternative for controlling false positives (Benjamini and Hochberg (1995), Dudoit *et al.* (2003)).

Facing the problem of possible dependent statistical tests, we are presently studying an approach to estimate the FDR and FWER in Algorithm 1 using again re-sampling data (Bickel (2002), Jain *et al.* (2005)). We give here a brief introduction to such an approach.

Let $V(c)$ be the average number of bootstrap Shapley value differences equal to or greater than c, in formula:

$$V(c) = \frac{1}{m} \sum_{r=1}^{m} card\Big(\{i \in N : \beta_i^r(\phi(\bar{v}_r^1), \phi(\bar{v}_r^2)) \geq c\}\Big), \tag{1}$$

with the convention that the cardinality of the empty set is zero, i.e. $|\emptyset| = 0$. Let $R(c)$ be the average number of observed Shapley value differences equal to or greater than c, in formula

$$R(c) = card\Big(\{i \in N : \delta_i(\phi(\bar{v}^1), \phi(\bar{v}^2)) \geq c\}\Big). \tag{2}$$

The simplest way to estimate FDR at the threshold value c is obtained via the following relation (Bickel (2002), Jain *et al.* (2005))

$$\widehat{FDR}(c) = \frac{V(c)}{R(c)}, \tag{3}$$

to control the estimated FDR at a level ϵ, let γ be the minimum value of $\delta_i(\phi(\bar{v}^1), \phi(\bar{v}^2))$ for which $\widehat{FDR}(\delta_i(\phi(\bar{v}^1), \phi(\bar{v}^2))) \leq \epsilon$ and reject the j-th null hypothesis if $\delta_i(\phi(\bar{v}^1), \phi(\bar{v}^2)) \geq \gamma$.

For what concerns controlling the FWER, as we already said different approach have been proposed. Here we present a single-step method to

adjust the p-values obtained in Algorithm 1 for controlling the FWER. For each $i \in N$, consider the adjusted p-value $\tilde{p}_i$ defined as follows

$$\tilde{p}_i = \frac{1}{m} card\Big(\{r \in \{1, \ldots, m\} : max_{j \in N}\big(\beta_j^r(\phi(\bar{v}_r^1), \phi(\bar{v}_r^2))\big) \geq \delta_i(\phi(\bar{v}^1), \phi(\bar{v}^2))\}\Big); \quad (4)$$

given the FWER α', reject the i-th null hypothesis if $\tilde{p}_i \leq \alpha'$.

On the other hand, the best method to use in order to control the FDR or the FWER in the CASh framework, where the interaction between genes is the goal of the analysis and test statistic independency cannot be assumed at all, has still to be identified and validated.

References

Amaratunga D., Cabrera J. (2004). Exploration and Analysis of DNA Microarray and Protein Array Data, Wiley-Interscience, New Jersey.

Benjamini Y., Hochberg Y. (1995). Controlling the false discovery rate: A practical and powerful approach to multiple testing. Journal of the Royal Statistical Society, Series B, 57:289-300.

Bickel, D. R. (2002). Microarray gene expression analysis:Data transformation and multiple comparison bootstrapping, Computing Science and Statistics 34, 383-400, Interface Foundation of North America (Proceedings of the 34th Symposium on the Interface, Montreal, Quebec, Canada, April 17-20, 2002)

Dudoit S., Yang Y., Speed T., Callow M. (2002). Statistical methods for identifying differentially expressed genes in replicated cDNA microarray experiments. Statistica Sinica, 12:111-139.

Dudoit S., Shaffer J.P., J.C. Boldrick (2003). Multiple hypothesis testing in microarray experiments, Statistical Science, 18(1), 71-103.

Efron B., Tibshirani R. J. (1993). An Introduction to the Bootstrap, Chapman & Hall/CRC: New York.

Jain N., Cho H.J., O'Connell M., Lee J.K. (2005) Rank-Invariant Resampling Based Estimation of False Discovery Rate for Analysis of Small Sample Microarray Data. BMC Bioinformatics, 6, 187:195.

Storey J.D., Tibshirani R. (2003) Statistical significance for genomewide

studies. Proceedings of the National Academy of Sciences of the United States of America, 100(16), 9440-9445.
