# Supplementary material for: Combining Shapley value and statistics to the analysis of gene expression data in children exposed to air pollution
Source: BMC Bioinformatics. 2008 Sep 2;9:361. doi: 10.1186/1471-2105-9-361 (PMC2556684; doi:10.1186/1471-2105-9-361)
Supplement: Additional file 3 — Example of calculations for CASh. contains an example of calculations for CASh on a toy expression matrix. [file 1471-2105-9-361-S3.pdf]

## Example of calculations for CASh

In this section we illustrate the intuition behind the Shapley value of microarray game on a numerical example. This example is also important to understand how CASh works in the analysis of gene expression differences between two conditions.

Consider the Boolean matrix $\bar{\mathbf{B}}^1$ of Table 1.(a) and the corresponding microarray game $(N, \bar{v}^1)$. Note that the characteristic function $\bar{v}^1$ assigns a number to all of the nonempty subsets of $N$, which are precisely $2^8 - 1 = 255$ coalitions of genes. We do not show all the values assumed by $\bar{v}^1$. As an example of computation of the characteristic function on a single coalition, consider coalition $\{1, 3, 8\}$. We have that $\bar{v}^1(\{1, 3, 8\}) = \frac{2}{7}$, since $\{1, 3, 8\}$ is a winning coalition two times out of the seven columns of $\bar{\mathbf{B}}^1$, precisely, on columns 1 and 7. The Shapley value of the microarray game $(N, \bar{v}^1)$ is reported in the second column of Table 2. Note that the most relevant gene according to the Shapley value $\phi(\bar{v}^1)$ is gene 1, followed by gene 5 and gene 7 with the same value and gene 3. Genes 4, 6, 8 and 2 close with the lowest Shapley values.

**(a)**

| | 1 | 2 | 3 | 4 | 5 | 6 | 7 |
|---|---|---|---|---|---|---|---|
| gene 1 | 1 | 0 | 0 | 0 | 0 | 0 | 1 |
| gene 2 | 0 | 0 | 0 | 1 | 0 | 1 | 0 |
| gene 3 | 0 | 0 | 0 | 1 | 1 | 1 | 1 |
| gene 4 | 0 | 0 | 0 | 0 | 1 | 1 | 0 |
| gene 5 | 0 | 0 | 1 | 1 | 0 | 0 | 0 |
| gene 6 | 0 | 0 | 0 | 0 | 1 | 1 | 0 |
| gene 7 | 0 | 1 | 0 | 1 | 0 | 0 | 0 |
| gene 8 | 0 | 0 | 0 | 0 | 0 | 1 | 1 |

**(b)**

| | 1 | 2 | 3 | 4 | 5 | 6 | 7 |
|---|---|---|---|---|---|---|---|
| gene 1 | 1 | 0 | 1 | 0 | 0 | 0 | 0 |
| gene 2 | 1 | 1 | 0 | 0 | 0 | 0 | 0 |
| gene 3 | 0 | 0 | 0 | 1 | 1 | 1 | 1 |
| gene 4 | 0 | 1 | 1 | 0 | 0 | 0 | 0 |
| gene 5 | 1 | 0 | 1 | 0 | 0 | 0 | 0 |
| gene 6 | 1 | 0 | 1 | 0 | 0 | 0 | 0 |
| gene 7 | 0 | 1 | 1 | 0 | 0 | 0 | 0 |
| gene 8 | 1 | 0 | 1 | 0 | 0 | 0 | 0 |

Table 1: (a) The Boolean matrix $\bar{\mathbf{B}}^1$. (b) The Boolean matrix $\bar{\mathbf{B}}^2$.

Now, consider the Boolean matrix $\bar{\mathbf{B}}^2$ of Table 1.(b), such that the $i$-th row is obtained as a permutations of the values in the $i$-th row in Table 1.(a), for each $i \in \{1, 2, 4, 6, 7, 8\}$. Note that only Boolean values for the third row in Table 1.(a) are not permuted in Table 1.(b). So, no gene-by-gene statistical analysis would be able to distinguish the behavior of gene 3 under the two conditions (a) and (b). On the other hand, in Table 1.(b), gene 3 plays a key role on samples $4, 5, 6$ and 7, where it is the unique gene with label 1. This point is well represented by the Shapley value, shown in the third column of Table 2. In fact, comparing the respective Shapley values for each gene

$i \in \{1, \ldots, 8\}$ in Tables 2, it is interesting to see that in the microarray game $\bar{v}^2$ only genes 2 and 3 increase their respective Shapley values with respect to microarray game $\bar{v}^1$. Gene 3 increases its Shapley value of about four times even if its row is exactly the same as in Table 1.(a). This increment is interesting in view of applying CASh.

Applying Algorithm 1 to the Boolean matrices $\bar{\mathbf{B}}^1$ and $\bar{\mathbf{B}}^2$ shown in Tables 1.(a) and 1.(b), respectively, with $\bar{\mathbf{B}}^1$ in the role of $\mathbf{B}^1$, $\bar{\mathbf{B}}^2$ in the role of $\mathbf{B}^2$ and with 1000 Bootstrap re-samples, we estimated the (un-adjusted for multiple comparisons) $p$-values presented in the fifth column of Table 2. At level 0.05, only the null hypothesis for gene 3, of no Shapley value difference between condition 1 and 2, can be rejected.

Note that gene 3 has exactly the same expression behavior under the two conditions 1 and 2, since the frequency of abnormal levels of expression for this gene is the same under the two conditions. Nevertheless, under condition 2, gene 3 is the only abnormally expressed gene on samples $4, 5, 6$ and 7. Consequently the role of gene 3 results very determinant in marking out the difference between the two conditions. This fact is represented by the strong difference of Shapley value of gene 3 between the two conditions, i.e. a difference which results statistically significant at $p < 0.05$ according to the test procedure introduced in Algorithm 1.

| gene $i$ | $\phi_i(\bar{v}^1)$ | $\phi_i(\bar{v}^2)$ | $\delta_i(\phi(\bar{v}^1), \phi(\bar{v}^2))$ | $p$-value |
|---|---|---|---|---|
| gene 1 | 0.19047619 | 0.05238095 | 0.13809524 | 0.336 |
| gene 2 | 0.06428571 | 0.07619048 | 0.01190477 | 0.857 |
| gene 3 | 0.15952381 | 0.57142857 | 0.41190476 | 0.039 |
| gene 4 | 0.07619048 | 0.07142857 | 0.00476191 | 0.933 |
| gene 5 | 0.17857143 | 0.05238095 | 0.12619048 | 0.407 |
| gene 6 | 0.07619048 | 0.05238095 | 0.02380953 | 0.691 |
| gene 7 | 0.17857143 | 0.07142857 | 0.10714286 | 0.458 |
| gene 8 | 0.07619048 | 0.05238095 | 0.02380953 | 0.706 |

Table 2: Column $\phi_i(\bar{v}^1)$ shows the Shapley value on the microarray game corresponding to the Boolean matrix in Table 1.(a); column $\phi_i(\bar{v}^2)$ shows the Shapley value on the microarray game corresponding to the Boolean matrix in Table 1.(b); column $\delta_i(\phi(\bar{v}^1), \phi(\bar{v}^2))$ shows the absolute difference $|\phi_i(\bar{v}^1) - \phi_i(\bar{v}^2)|$; column $p$-value shows the un-adjusted $p$-values obtained by Algorithm 1 applied to the Boolean matrices $\bar{\mathbf{B}}^1$ and $\bar{\mathbf{B}}^2$.
